# A FIN-LDMOS with Bulk Electron Accumulation Effect

**DOI:** 10.3390/mi14061225

**Published:** 2023-06-10

**Authors:** Weizhong Chen, Zubing Duan, Hongsheng Zhang, Zhengsheng Han, Zeheng Wang

**Affiliations:** 1 College of Electronics Engineering, Chongqing University of Posts and Telecommunications, Chongqing 400065, China; chenwz@cqput.edu.cn (W.C.); zhanghs@cqupt.edu.cn (H.Z.); 2 Institute of Microelectronics, Chinese Academy of Sciences, Beijing 100029, China; 3 CSIRO Manufacturing, 36 Bradfield Road, P.O. Box 218, Lindfield, NSW 2070, Australia

**Keywords:** bulk electron accumulation (BEA), extended superjunction trench gate, extended drain (ED), *BV* and *R*_on,sp_

## Abstract

A thin Silicon-On-Insulator (SOI) LDMOS with ultralow Specific On-Resistance (*R*_on,sp_) is proposed, and the physical mechanism is investigated by Sentaurus. It features a FIN gate and an extended superjunction trench gate to obtain a Bulk Electron Accumulation (BEA) effect. The BEA consists of two p-regions and two integrated back-to-back diodes, then the gate potential *V*_GS_ is extended through the whole p-region. Additionally, the gate oxide *W*_oxide_ is inserted between the extended superjunction trench gate and N-drift. In the on-state, the 3D electron channel is produced at the P-well by the FIN gate, and the high-density electron accumulation layer formed in the drift region surface provides an extremely low-resistance current path, which dramatically decreases the *R*_on,sp_ and eases the dependence of *R*_on,sp_ on the drift doping concentration (*N*_drift_). In the off-state, the two p-regions and N-drift deplete from each other through the gate oxide *W*_oxide_ like the conventional SJ. Meanwhile, the Extended Drain (ED) increases the interface charge and reduces the *R*_on,sp_. The 3D simulation results show that the *BV* and *R*_on,sp_ are 314 V and 1.84 mΩ∙cm^−2^, respectively. Consequently, the *FOM* is high, reaching up to 53.49 MW/cm^2^, which breaks through the silicon limit of the RESURF.

## 1. Introduction

The Lateral Double-diffused Metal–Oxide Semiconductor (LDMOS) is a very important device in power-integrated circuits and electronic power systems [1,2,3], which are used in many places in our daily life. The Breakdown Voltage (*BV*) and the Specific On-Resistance (*R*_on,sp_) are significant parameters to evaluate the quality of devices [4,5,6,7]. For the conventional LDMOS, there is an unavoidable trade-off relationship between the *BV* and *R*_on,sp_, which can be written as *R*_on_ ∝ *BV*^2.5^, while what we need are high *BV* and low *R*_on,sp_. Baliga’s Figure Of Merit (*FOM*) is calculated by *BV*^2^/*R*_on,sp_ to evaluate the device, where a higher value is better [8,9,10]. Many advanced theories and structures have been investigated to increase the *FOM* of the power devices [11,12,13,14]. For example, for the BFG LDMOS proposed in [11], the author made half of the device into a grid, and for the HKGF LDMOS proposed in [14], the authors distinguished the device drift into three parts, each surrounded by a three-dimensional High-K dielectric, both of which greatly enhanced the control ability of the device and greatly reduced the *R*_on,sp_ of the device. The Enhanced Dielectric layer Field (ENDIF) theory can introduce a higher electric field between the top layer of silicon and the buried oxygen layer, which can obviously enhance *BV* [15,16,17]. However, it is possible to increase *R*_on,sp_ with the application of ENDIF theory. For example, the T-SJ LDMOS proposed in [17] enhances the *BV* of the device by making the top layer of silicon near the drain extremely thin, but greatly reduces the volume of the device drift region, thus reducing the *R*_on,sp_ of the device. The SuperJunction (SJ) structure can effectively increase the drift doping concentration by using N-type semiconductors and P-type semiconductors to assist each other; thus, the *R*_on,sp_ is reduced and the BV is guaranteed simultaneously, and thus the *FOM* can be effectively improved [18,19,20,21]. However, the superjunction structure is often placed on the P-type substrate in lateral devices, and the surface superjunction region is affected by the Substrate-Assisted Depletion (SAD) effect, which results in reduced pressure tolerance. Moreover, the power FINFET with a 3D electron channel and the LDMOS with an Accumulation Extended Gate (AEG LDMOS) are also used to improve the device [22].

In this paper, the FIN-LDMOS with Bulk Electron Accumulation (BEA-LDMOS) is first proposed. The device adopts an SOI structure, which can effectively suppress the SAD effect. The bulk electron accumulation effect produced by the extended superjunction trench gate in the N-drift and the ENDIF effect produced by the Extended Drain are produced. Moreover, the assisted depletion effect is performed by the extended superjunction trench gate, which dramatically reduces the *R*_on,sp_ while *BV* is guaranteed. The devices are performed by Synopsys Sentaurus, and the main physics models are as follows: Effectice Intrinsic Density, Mobility (High Field Saturation Enormal PhuMob DopingDependence), and Recombination (SRH Auger Avalanche) [23].

## 2. Device Structure and Mechanism

### 2.1. Device Structure of the BEA

**Figure 1:**
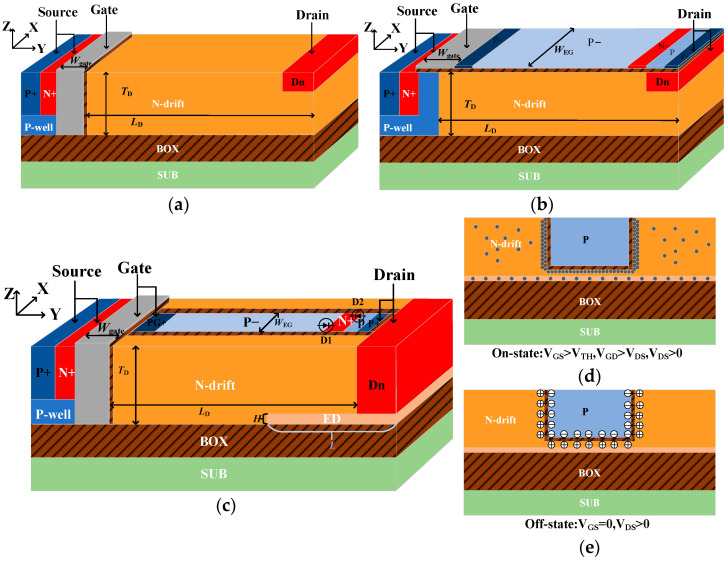
The three-dimensional (3D) schematic and mechanism of the three proposed devices. (**a**) FIN-LDMOS, (**b**) AEG-LDMOS, (**c**) BEA-LDMOS, (**d**) the Bulk Electron Accumulation (BEA) effect induced at the N-drift, (**e**) the assisted depletion between the P-region and N-drift in the Off-state. Diode D1 is formed by the (P−/N+) and D2 is formed by the (P/N+) junction.

The FIN-LDMOS, AEG-LDMOS, and the proposed BEA-LDMOS are compared together in Figure 1. The FIN-LDMOS greatly increases the channel area of the device by turning the one-dimensional gate into a three-dimensional one, to reduce the *R*_on,sp_. The AEG-LDMOS structure is characterized by extending the gate, which is only near the source region in the conventional LDMOS, to the drain, separated by SiO_2_. In order to extend the drain potential better, two back-to-back PN junctions are used in the extension gate to generate a charge accumulation effect at the top of the N-drift area, forming a low-resistance channel and greatly reducing the *R*_on,sp_ of the device. However, the *BV* will be affected by the PN junction in the extension grid, which reduces the *BV* of the device. The 3-D structure of the BEA-LDMOS is shown in Figure 1. The extended superjunction trench gate is formed as follows: the two back-to-back diodes are introduced at the collector, and the P-region and N-drift are separated by the SiO_2_. Moreover, the two ends of the P-region are, respectively, shortly connected to the gate electrode and the drain electrode, as shown in Figure 1a. When the device is in the on-state, the positive *V*_GS_ is extended through most of the P-region to the positively biased D_1_, and positive *V*_DS_ is extended through the P-region to the positively biased D_2_. Thus, the P-region is covered by the *V*_GS_ and *V*_DS_; then, a Bulk Electron Accumulation (BEA) is generated in the drift region, as shown in Figure 1b, and the metal–insulator–semiconductor structure is composed of P-region/oxide/N-drift. It is equivalent to a low-resistance 3-D channel in the drift region. Therefore, the *R*_on,sp_ of the device is greatly reduced. Figure 1c indicates the breakdown mechanism of the BEA-LDMOS in the off-state, and it is similar to the conventional superjunction. The P-region and the N-drift region are separated by SiO_2_, and there is still a built-in electric field from the N-drift region toward the P-region, resulting in the formation of a depletion region near SiO_2_. Therefore, the electric field of the N-drift is modulated, thus helping to increase *BV*. At the same time, the Extended Drain structure is also introduced in the drift area, which can not only introduce the high electric field near the drain region into the more voltage-resistant silicon dioxide buried layer to increase the *BV* of the device, but also play the role of low-resistance channel, reducing the *R*_on,sp_ of the device. The key parameters of the devices are listed in Table 1, with a drift length L_D_ of 21.0 μm, depth *T*_D_ of 5.0 μm, Extended Drain length *L* of 9 μm, and thickness of 0.4 μm. The optimized drift doping *N*_drift_ of 2.2 × 10^15^ cm^−3^ is designed for the BEA-LDMOS.

**Figure 2:**
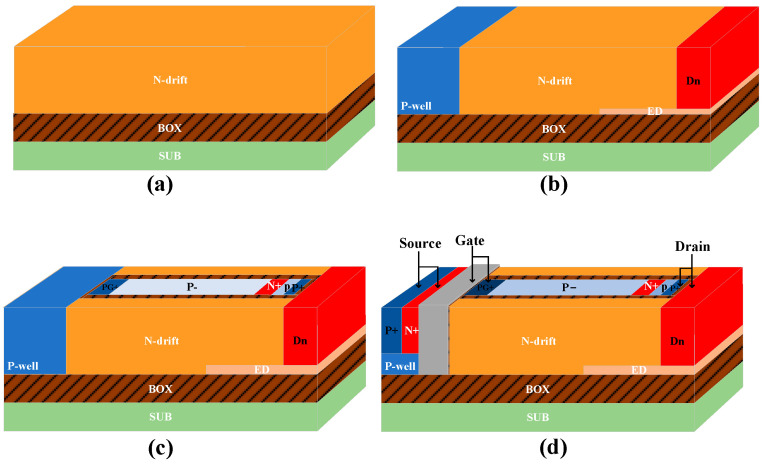
Simplified key process of the proposed BEA-LDMOS. (**a**) SOI substate, (**b**) Ion doping, (**c**) Thermal oxidation, (**d**) Electrode deposition.

The simplified production process of the BEA-LDMOS is given as follows: The emphasis is the extended superjunction trench gate and Extended Drain. The SOI wafer in Figure 2a is injected with oxygen ions and annealed to form a silicon dioxide isolation layer. The P-well and Extended Drain are obtained by implantation, as shown in Figure 2b. The following undergo thermal oxidation to grow the isolation layer and undergo secondary ion implantation doping, as shown in Figure 2c. The subsequent processes such as metallization and passivation are compatible with conventional LDMOSs in Figure 2d.

### 2.2. Mainly Applied Physical Models

The main physical models used in this simulation include the carrier mobility model, carrier recombination model, and avalanche breakdown generation model [24,25,26]. Carrier mobility is expressed as follows:

(1) $$\frac{1}{\mu }=\frac{\exp(-\frac{x}{l_{\mathrm{cirt}}})}{\mu_{\mathrm{ac}}}+\frac{\exp(-\frac{x}{l_{\mathrm{cirt}}})}{\mu_{\mathrm{sr}}}+\frac{1}{\mu_{b}}+\frac{1}{\mu_{F}}$$

where *μ*_ac_ represents the surface phonon scattering model, *μ*_sr_ represents the surface roughness scattering model, *x* represents the distance between the insulator and the semiconductor interface, *μ_b_* represents the low-field-mobility model, and *μ_F_* represents the high-field-mobility model.

In the simulation, we use the SRH carrier recombination model, which can accurately simulate the recombination mechanism of carrier under quantum effects. The carrier recombination model is expressed as follows:

(2a) $$R_{net}^{SRH}=\frac{np-\gamma_{n}\gamma_{p}n_{i,eff}}{T_{p}(n+\gamma_{n}n_{i,eff})+T_{n}(p+\gamma_{p}n_{i,eff})}$$

(2b) $$\gamma_{n}=\frac{n}{N_{C}}\exp(-\frac{E_{Fn}-E_{C}}{kT_{n}})$$

(2c) $$\gamma_{p}=\frac{p}{N_{V}}\exp(-\frac{E_{V}-E_{Fp}}{kTp})$$

In the formula, *T_n_* represents the lifetime of non-equilibrium minority electron, *T_p_* represents the lifetime of non-equilibrium minority hole, *N_C_* represents the effective state density of the conduction band, *N_V_* represents the effective state density of the valence band, *E_Fn_* represents the quasi-Fermi level of the conduction band, *E_Fp_* represents the quasi-Fermi level of the valence band.

In order to accurately simulate the breakdown voltage of the device, the avalanche breakdown generation model is introduced in the simulation process. When the device is working in the blocking voltage, as the drain voltage continues to increase, the internal electric field of the device becomes stronger. When the maximum electric field inside the device is greater than or equal to the critical breakdown electric field of silicon, the charge multiplication effect will occur, and the leakage of the device will increase sharply, resulting in electrical breakdown of the device. The avalanche breakdown generation model is expressed as follows:

(3a) $$G^{Avalanche}=\alpha_{n}nv_{n}+\alpha_{p}pv_{p}$$

(3b) $$\alpha =\gamma ae^{-\frac{\gamma b}{F}}$$

(3c) $$\gamma =\frac{\tan h(\frac{h\omega_{op}}{2kT_{0}})}{\tan h(\frac{h\omega_{op}}{2kT})}$$

In the formula, *α* is the ionization factor, it’s the inverse of the mean free path, *F* represents the Vector mechanics, $h\omega_{op}$ represents the optical phonon energy, *y* represents the Dependence coefficient of phonon.

## 3. Results and Discussion

### 3.1. Control Mechanism and Bulk Electron Accumulation Effect of the BEA

**Figure 3:**
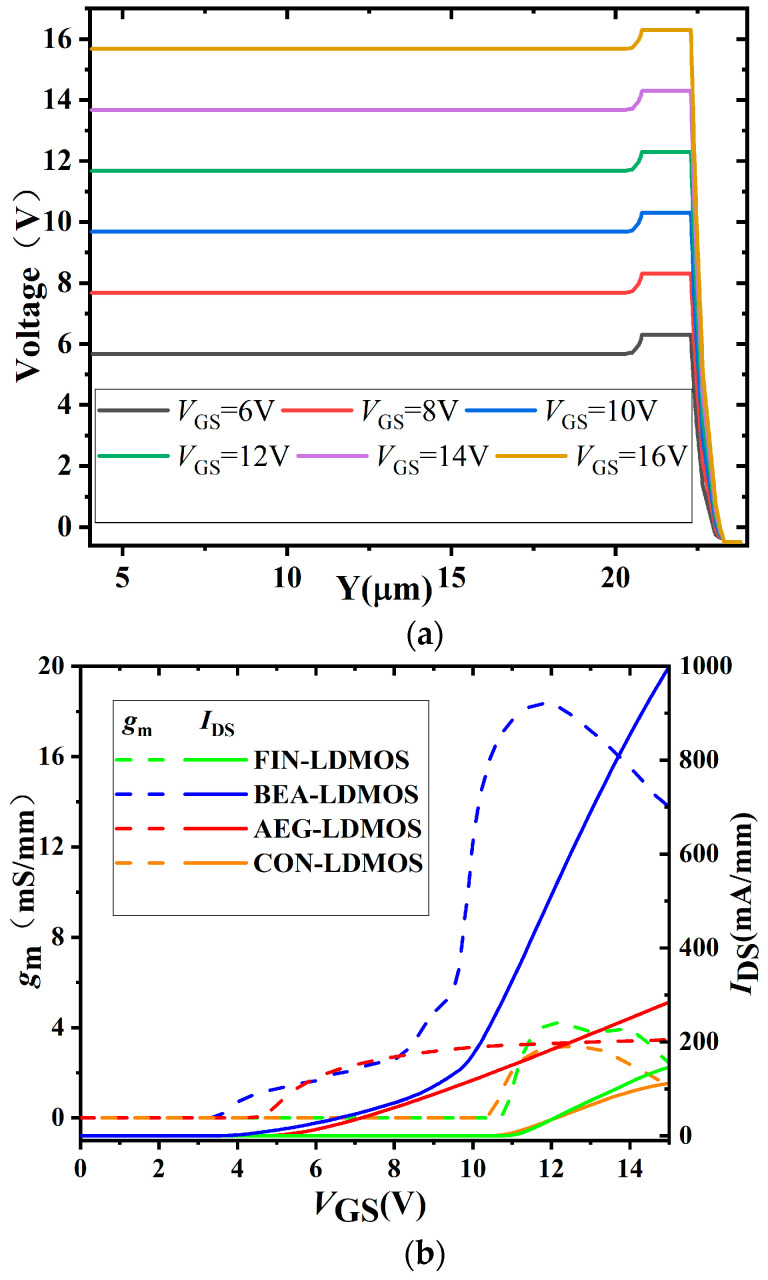
The gate potential *V*_GS_ in the on-state, transfer, and *g*_m_ characteristics for the devices. (**a**) Potential distribution along the p–n–p for the BEA-LDMOS. (**b**) Transfer and *g_m_* of the CON-LDMOS, FIN-LDMOS, AEG-LDMOS, and BEA-LDMOS.

Figure 3 shows the transfer, transconductance (*g*_m_), and gate potential characteristics for the devices. Figure 3a shows that when *V*_GS_ is increased from 6 V to 16 V, and the *V*_DS_ of the drain is grounded, *V*_GS_ is extended through the whole P-region, and it is shunted by the negatively biased D_2_. Figure 3b compares the transfer and *g*_m_ characteristics for the CON-LDMOS, AEG-LDMOS, FIN-LDMOS, and BEA-LDMOS. The peak *g*_m_ for the devices is 2.26, 3.16, 4.21, and 18.33 mS/mm, respectively. Because the higher peak *g*_m_ can be achieved by the 3-D bulk electron channel, the BEA shows the best control capability of *I*_DS_.

**Figure 4:**
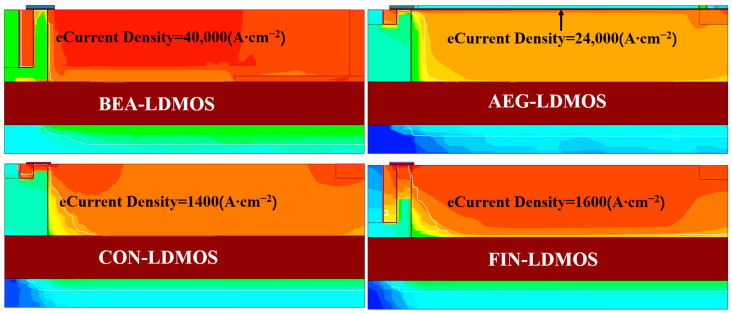
The electgron Current density distribution for the four devices at the On-state.

Figure 4 shows the electron current densities of devices in the on-state. It can be seen that the electron current density of the AEG-LDMOS and BEA-LDMOS are much higher than those of the other two devices due to the existence of a charge accumulation effect. Moreover, because of the 3-D charge accumulation effect of the BEA-LDMOS, while the charge accumulation effect of the AEG is one-dimensional, the area of the low-resistance channel formed by the BEA-LDMOS is much higher than that of AEG-LDMOS, so the electron current density of the BEA-LDMOS is the largest of all four devices.

**Figure 5:**
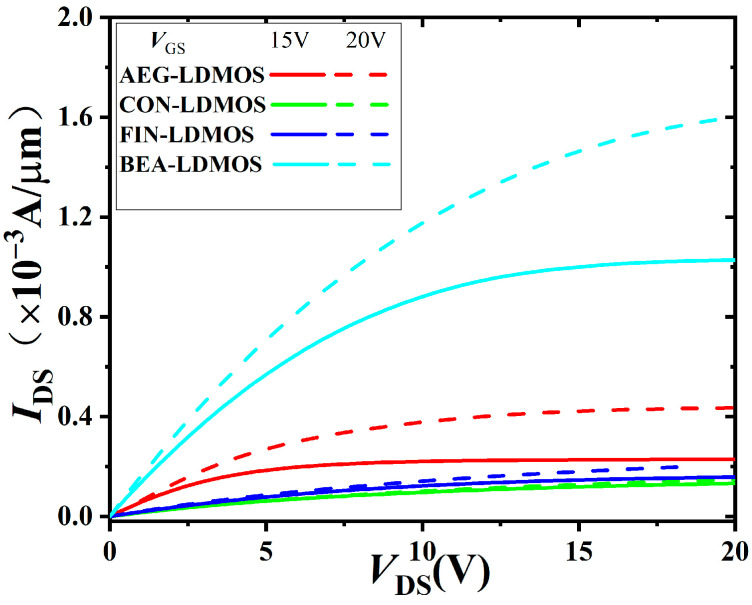
Output characteristics of the devices, and *V*_GS_ of 15 and 20 V are applied.

Figure 5 shows the output *I*_DS_-*V*_DS_ characteristics of the four devices at the forward conduction. For the proposed BEA-LDMOS, the P-region is covered by *V*_GS_ and *V*_DS_ to obtain a 3-D low-resistance channel, so the linear current and saturation current in the drift region are much higher than those of the CON-LDMOS, AEG-LDMOS, and FIN-LDMOS under the same *V*_DS_. Thus, stronger conductivity and ultra-low *R*_on,sp_ are achieved.

### 3.2. Specifics of Resistance R_on,sp_ and Breakdown Voltage BV

**Figure 6:**
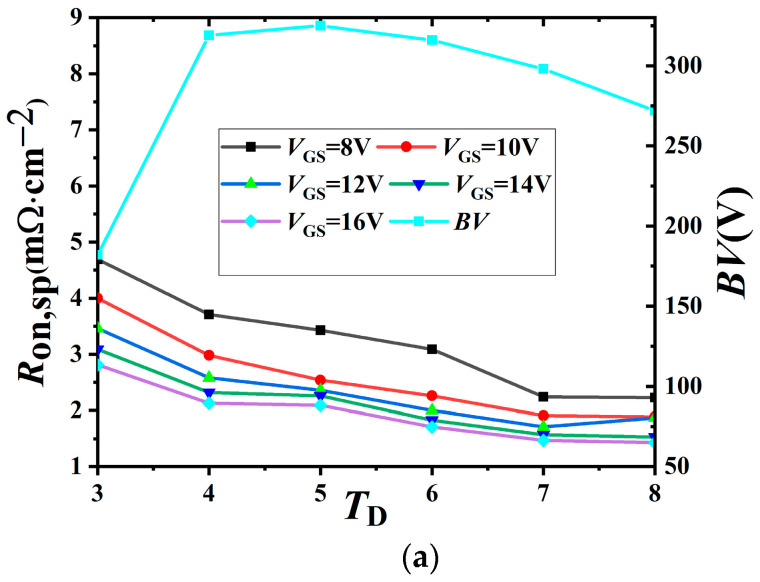

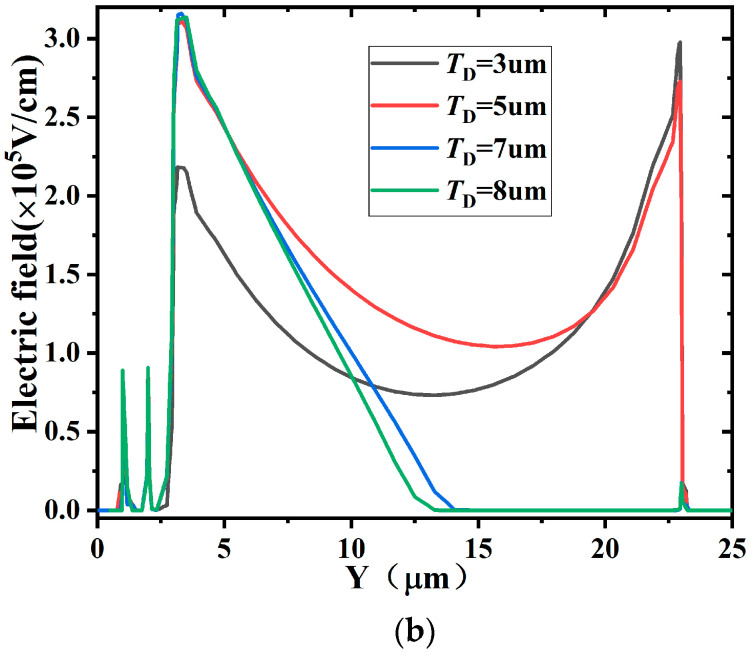
Influence of key parameter *T*_D_ on the *R*_on,sp_, *BV*, and electric field for the BEA-LDMOS (*T*_D_ is the thickness of the N-drift). (**a**) Influence on the *R*_on,sp_ and *BV*, (**b**) influence on the electric field of top layer.

Figure 6 shows the influence of the thickness of the top layer of silicon on *R*_on, sp_ and *BV* of the device. It can be seen from the figure that *R*_on,sp_ gradually decreases with the increase in *T*_D_ under different gate voltages. This can be explained by the formula for volume resistance:

(4a) $$R=R_{s}\frac{L}{W}$$

(4b) $$R_{s}=\frac{\rho }{T_{D}}$$

where *L* and *W* are the length and width of the device channel, respectively; *R_s_* is the resistance of the block; ρ is the resistivity; and *T*_D_ is the thickness of the silicon film. However, the *BV* first increases and then decreases with the increase in *T*_D_, and the optimum *BV* is 314 V when *T*_D_ is 5.0 μm. This is because when *T*_D_ is small, the longitudinal breakdown voltage of the device is very low, and the *BV* of the device mainly depends on the longitudinal breakdown voltage. When *T*_D_ is too large, the relationship between *N*_drift_ and *T*_D_ does not conform to the RESURF theory [27], the device will breakdown in advance, as shown in Figure 6b, and the electric field will be 0 at half of the drift area of the device.

**Figure 7:**
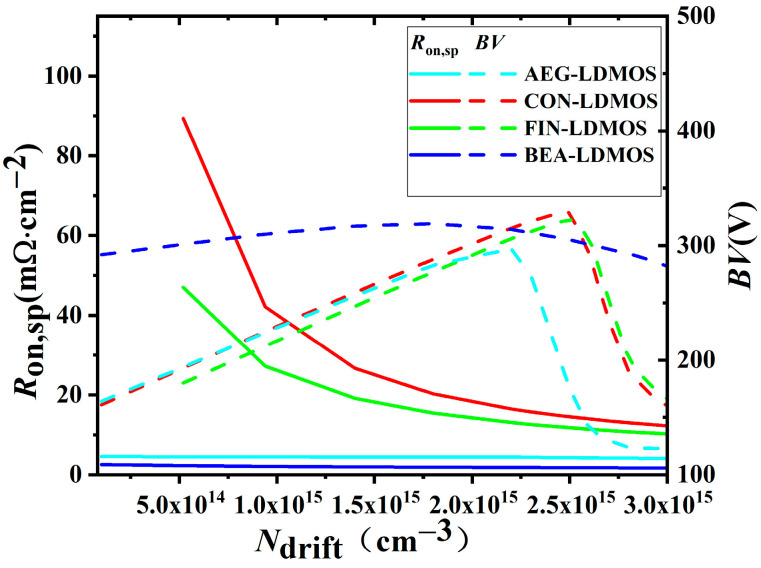
Effect of doping concentration of N-drift region on *BV* and *R*_on,sp_ for the four devices.

Figure 7 shows the influences of the doping *N*_drift_ on the *R*_on,sp_ and *BV* for the devices. For the CON-LDMOS, the optimized *BV* and *R*_on,sp_ are 329 V and 14.54 mΩ∙cm^2^ when the *N*_drift_ is 2.5 × 10^15^ cm^−3^, respectively. For the FIN-LDMOS, the optimized *BV* and *R*_on,sp_ are 323 V and 11.78 mΩ∙cm^2^ when the *N*_drift_ is 2.5 × 10^15^ cm^−3^, respectively. For the BEA-LDMOS, the optimized *BV* and *R*_on,sp_ are 314 V and 1.84 mΩ∙cm^2^ when the *N*_drift_ is 2.2 × 10^15^ cm^−3^, respectively. It can be seen that the *BV* of the four devices increases first and then decreases with the increase in *N*_drift_. This is because when the doping concentration is very low, the maximum electric field of the device is near the drain region, and the breakdown voltage of the device mainly depends on the heterojunction formation of the drain region and N-drift region. When the doping concentration in the N-drift region gradually increases, the PN junction formed in the drift region and the P-well also begins to participate in the voltage resistance. When the N-drift region doping concentration continues to increase, the maximum electric field of the device will appear near the source region, and the doping concentration difference between the drain and the drift region is very small. The breakdown voltage of the device mainly depends on the PN junction formed by the drift region and P-well. The breakdown voltage reaches its maximum when the two electric field spikes are almost high. The *R*_on,sp_ of CON-LDMOS and FIN-LDMOS decreases obviously with the increase in *N*_drift_, but the *R*_on,sp_ of AEG-LDMOS and BEA-LDMOS almost does not change with the change in *N*_drift_. This is because these two devices have low-resistance channels formed by the electron accumulation effect, so *R*_on,sp_ does not depend on *N*_drift_. Consequently, Baliga’s Figures OF Merit (FOMs) of the CON-LDMOS, FIN-LDMOS, AEG-LDMOS, and BEA-LDMOS are calculated as 7.42 MW/cm^2^, 8.86 MW/cm^2^, 20.02 MW/cm^2^, and 53.43 MW/cm^2^, respectively.

**Figure 8:**
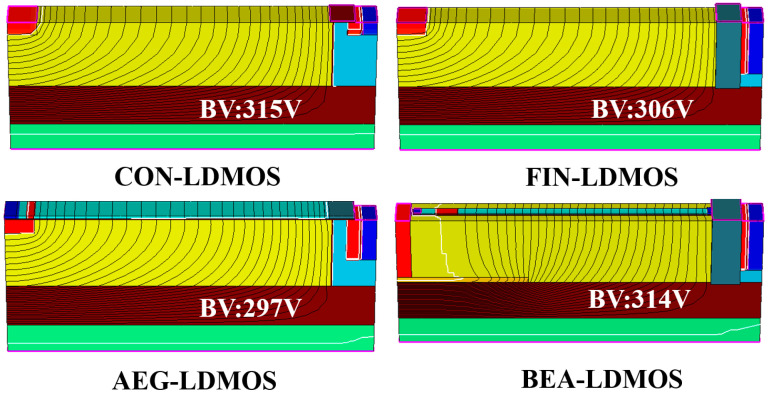
The 3-D equipotential contours at the avalanche breakdown at the same *N*_drift_.

Figure 8 shows the corresponding equipotential distribution at the avalanche breakdown for the four devices. The yellow area in the figure is the N-drift area, the brown area is the buried oxygen layer, the green area is the P-type substrate, the top left is the drain, the right is the source and the gate. It is noted that the BVs of the CON-LDMOS, FIN-LDMOS, AEG-LDMOS, and BEA-LDMOS are 315, 306, 297, and 314 V, respectively. The CON-LDMOS, FIN-LDMOS, and AEG-LDMOS have a similar distribution of potential lines, but there is no distribution of potential lines in the area near the drain of the BEA-LDMOS. This is because the Extended Drain introduces the higher electric field into the silicon dioxide layer, so the silicon dioxide layer below the Extended Drain has a denser distribution of the potential line. Because SiO_2_ has a smaller interfacial defect density and a larger dielectric constant than silicon, SiO_2_ can withstand a higher voltage and can improve the *BV* of the device.

**Figure 9:**
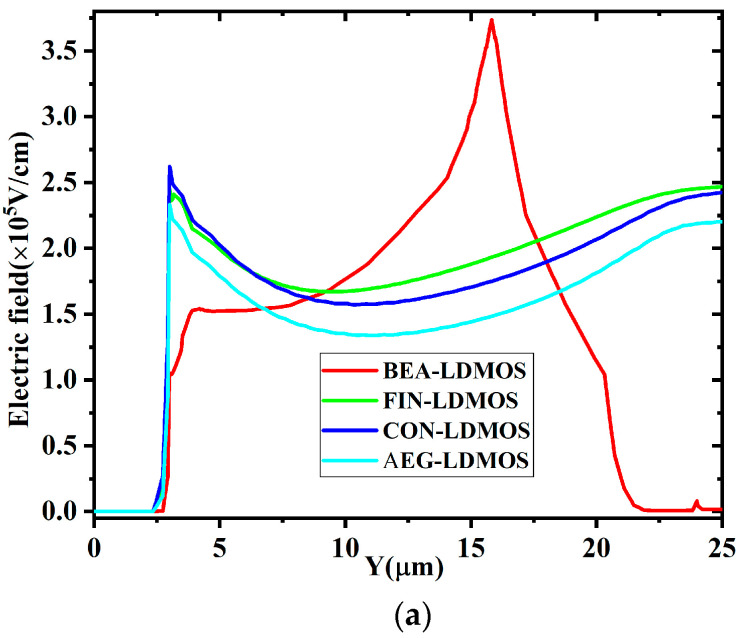

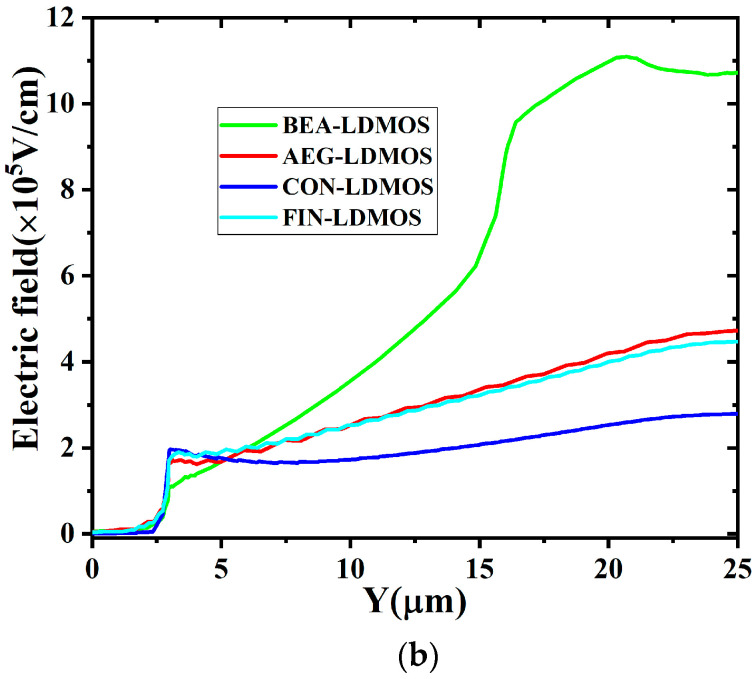
The electric field distribution at the avalanche breakdown at (**a**) X = 1.9 μm, Z = 6 μm; (**b**) X = 1.9 μm, Z = 4.9 μm.

Figure 9 demonstrates the electric field Efield distribution along the cut line of X = 1.9 μm, Z = 6 μm and X = 1.9 μm, Z = 4.9 μm. It is noted that the Efield in the N-drift of the BEA-LDMOS reaches a maximum at the end of the Extended Drain and then drops rapidly, as shown in Figure 9a. This is because the Extended Drain of high doping forms a heterojunction with the drift region of low doping concentration. In heterogeneous junctions, electrons and holes are unevenly distributed on both sides due to different material doping concentrations. Since the concentration of electrons in the highly doped region is higher than that in the low-doped region, electrons will diffuse from the high-doped region to the low-doped region, while holes will diffuse from the low-doped region to the high-doped region. Electrons and holes will meet at the center of the junction, resulting in a large number of recombination. This recombination results in a region of space charges near the junction, and the uneven distribution of charges in this region leads to the formation of a sharp electric field. Meanwhile, the Efield in the buried silicon dioxide layer of the BEA-LDMOS is much higher than those of the other three devices, as shown in Figure 9b. This is because the Extended Drain draws the high electric field in the N-drift region into the buried silicon dioxide layer, which can withstand higher voltages.

### 3.3. Influence of Unique Key Parameters on the R_on,sp_, Peak g_m_, and BV of the BEA LDMOS

**Figure 10:**
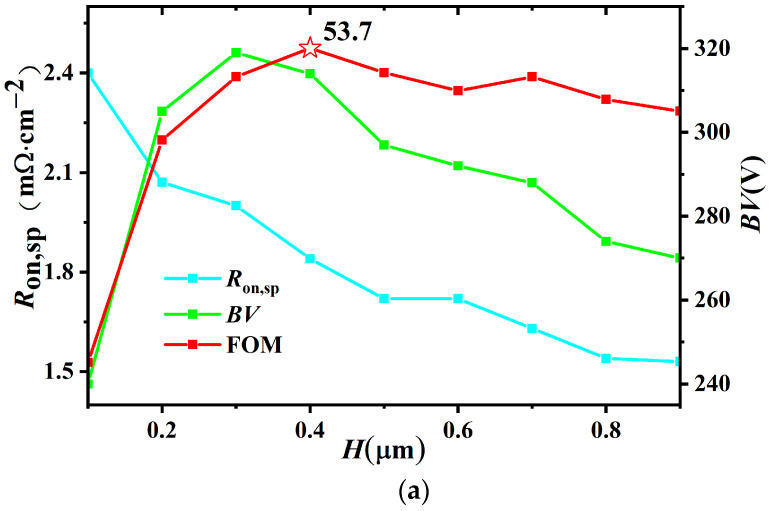

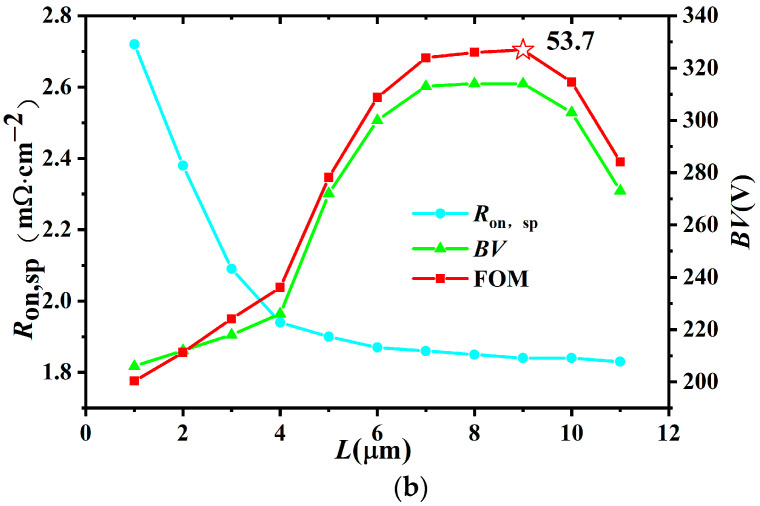
Key parameters: (**a**) thickness (*H*) of the Extended Drain, (**b**) length (*L*) influence on the *R*_on,sp_, peak *g*_m_, and *BV* for the BEA-LDMOS.

Figure 10 shows the influence of the length (*L*) and thickness (*H*) of the Extended Drain on the *R*_on,sp_ and *BV* for the BEA-LDMOS. In Figure 10a, the Extended Drain is equivalent to a low-resistance channel, so increasing the thickness of the Extended Drain is equivalent to increasing the volume of the low-resistance channel. Consequently, the specific conduction resistance decreases with increase in thickness of the Extended Drain. In Figure 10b, the specific conduction resistance decreases with increase in the length of the Extended Drain. The *BV* of the device generally increases first and then decreases with the increase in the thickness and length of the Extended Drain. This is because at the beginning, with the increase in the volume of the Extended Drain, the high electric field can be better introduced into the silicon dioxide layer. However, when the increase exceeds a certain range, it is equivalent to increasing the drift concentration, which will reduce the breakdown voltage. Considering the trade-off property between the *R*_on,sp_ and *BV*, the thickness of 0.4 μm and length of 9 μm are selected as the best parameters.

### 3.4. Dynamic Characteristics

**Figure 11:**
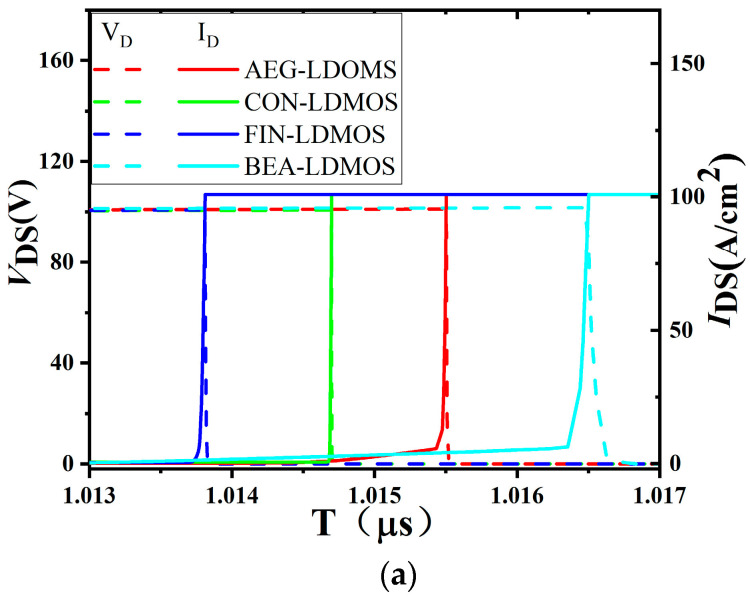

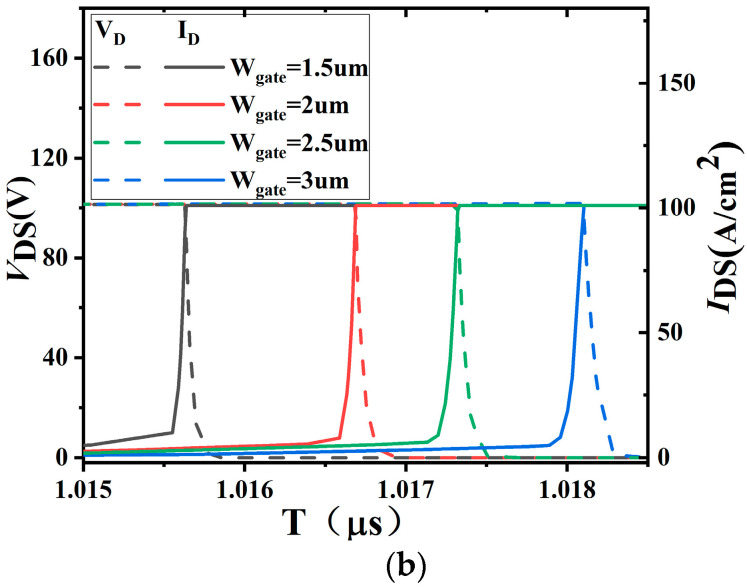
(**a**) Capacitance switching characteristics of the four devices. Turn-on and turn-off curves under inductive load.(**b**) Switching characteristic curves at different gate widths.

The switching characteristics under inductive load are shown in Figure 11a, and a slower turn-on speed *T*_ON_ and turn-off speed *T*_OFF_ of the BEA-LDMOS are observed compared to the CON-LDMOS, AEG-LDMOS, and FIN-LDMOS. Because the gate capacity is proportional to the gate area, the larger the area, the larger the gate capacity. The BEA-LDMOS not only has a FIN structure, but also has an extended superjunction trench gate, so the gate area is much larger than those of the other three devices. Figure 11b shows the effect of different widths of the gate on the switching speed of the device. It can be seen that the wider the gate, the lower the switching speed of the device. The switching performance of the device can be improved by reducing the width of the gate.

### 3.5. The Trade-Off Property between the R_on,sp_ and BV

**Figure 12:**
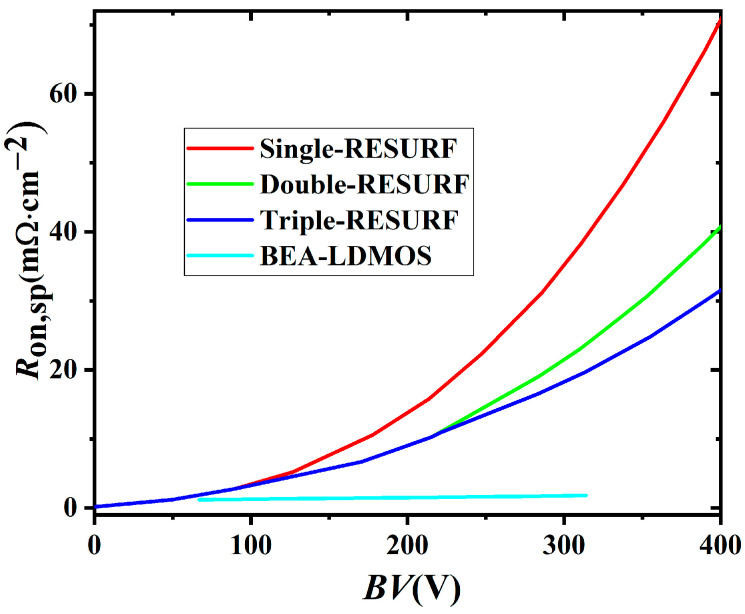
The trade-off relationship between *R*_on,sp_ and *BV* for the BEA-LDMOSs and the RESURF. The *FOM* is calculated by the *FOM* = *BV*^2^/*R*_on,sp_.

Figure 12 demonstrates the trade-off characteristic and FOM for the BEA LDMOS, single RESURF, double RESURF, and triple RESURF in Ref. [9], which are the classic three structures. It can be seen from the figure that the *R*_on,sp_ of the device is still very low at a larger *BV*. According to *FOM* = *BV*^2^/*R*_on,sp_, it can be concluded that the *FOM* of BEA is largest and achieves the best trade-off property. The main performance indexes of the four devices compared in this paper are shown in Table 2.

## 4. Conclusions

The mechanism and electric characteristics of the BEA-LDMOS are proposed and researched. The *V*_GS_ of the BEA is extended through the P-region, and the full buck accumulation effect is formed at the inside of the N-drift, where a 3-D low-resistance channel at the N-drift is achieved. In addition, the Extended Drain is also equivalent to a low-resistance channel. Thus, the *R*_on,sp_ is significantly decreased. Simultaneously, the superior *BV* is guaranteed by the charge compensation and assisted depletion effect between the P-type doping and N-drift. Consequently, a *FOM* of 53.49 MW/cm^2^ is achieved, which breaks through the silicon limit of the RESURF.

## Figures and Tables

**Table 1:** Key parameters used in simulation.

Symbol	Description	FIN-LDMOS	CON-LDMOS	AEG-LDMOS	BEA-LDMOS
*L*_D_	drift length (μm)	21	21	21	21
*T*_D_	drift depth (μm)	5	5	5	5
*W_gate_*	Gate wideth (μm)	2	2	2	2
*N*_drift_	N-drift doping (cm^−3^)	2.5 × 10^15^	2.5 × 10^15^	2.5 × 10^15^	2.5 × 10^15^
*h-top*	AEG structure thickness (μm)	--	--	0.2	--
*W*_oxide_	Gate oxide width (μm)	0.1	0.1	0.1	0.1
*W*_EG_	Extended gate width (μm)	--	--	2.2	0.6
*L_FIN_*	FIN-Gate length (μm)	1	--	--	1
*L*	Extended drain lenth (μm)	--	--	--	9
*H*	Extended drain thickness (μm)	--	--	--	0.4

**Table 2:** Trade-off Property between the *R*_on,sp_ and *BV*.

Symbol	FIN-LDMOS	CON-LDMOS	AEG-LDMOS	BEA-LDMOS
*BV* (V)	323	329	297	314
*R*_on,sp_ (mΩ∙cm^−2^)	11.78	14.54	4.41	1.84
*FOM*	8.86	7.42	20.01	53.43
*N*_drift_ (cm^−3^)	2.5 × 10^15^	2.5 × 10^15^	2.5 × 10^15^	2.5 × 10^15^

## Data Availability

Data is unavailable due to privacy or ethical restrictions.
